# Phenotypic and Clinical Traits That Correlate with Cognitive Impairment in Caucasian Females

**DOI:** 10.1089/whr.2021.0007

**Published:** 2021-11-23

**Authors:** Colleen Reisz, Karen Figenshau, An-Lin Cheng, Abdelmoneim Elfagir

**Affiliations:** ^1^ Department of Medicine and University of Missouri Kansas City School of Medicine, Kansas City, Missouri, USA.; ^2^ Department of Biomedical and Health Informatics, University of Missouri Kansas City School of Medicine, Kansas City, Missouri, USA.

**Keywords:** Alzheimer's dementia, dementia, estrogen, fetal origins, Fitzpatrick skin phototype, hippocampus, prenatal stress

## Abstract

***Background:*** Dementia affects more women than men. This suggests sex steroid-dependent structural and functional differences between male and female brains. Natural and iatrogenic changes to women's reproductive health may correlate with risk for dementia.

***Objective:*** To identify surrogate markers of key transitions within the reproductive axis that could correlate with dementia pathology in women.

***Specific Research Question:*** Could examination of the reproductive axis from birth to senescence expand our understanding of the gender predominance of dementia in women? Proxy measurements for fetal origins, reproduction, and age-related effects on estrogen-dependent tissues were collected to study dementia risk in women.

***Methods:*** Deidentified data were collected from 289 older Caucasian female patients from an out-patient clinic in Kansas City, Missouri. Women patients 65 years and older were offered the opportunity to join the study and written consent was obtained from all participants. Data were collected from 2017 to 2019.

***Results:*** Our subjects ranged in age from 65 to 98 years old, with a mean of 76 years old. Spearman correlation analysis showed significant correlation between dementia status and age (*r* = 0.219, *p* = 0.000), Fitzpatrick skin phototype (*r* = −0.141, *p* = 0.019), birth order (*r* = 0.151, *p* = 0.028), current height as measured in the office (*r* = −0.215, *p* = 0.001), and maximum height per patient recall (*r* = −0.173, *p* = 0.005). Results from the logistic regression model show that specific predictors of risk for dementia were age (odds ratio [OR] = 1.082 [1.034–1.132]; *p* = 0.0007), Fitzpatrick skin phototype 1 versus 3 (OR = 8.508 [1.075–67.313]; *p* = 0.0227), and current height (OR = 0.766 [0.642–0.915]; *p* = 0.0032). Of the four variables related to fetal origins: maternal age, number of siblings, birth order, and age difference between the subject and the next older sibling, none were found to be statistically significant. Since age is a significant predictor of risk for dementia, it was included as a covariate in the aforementioned logistic regression models.

***Conclusions:*** Our results showed that dementia in Caucasian women was associated with age, lower Fitzpatrick phototype, and current height. Dementia-related pathological processes in the brain may accrue over a woman's lifetime.

## Introduction

Dementia is a common progressive neurodegenerative disease and a leading cause of disability and poor health among the geriatric population. With the increasing age of the population, it is estimated that 14 to 16 million Americans will be diagnosed with some form of dementia by 2050, unless new interventions to prevent or delay the onset of disease are identified.^1^ The rising incidence of dementia, including Alzheimer's disease (AD), is of particular concern to women, because it is estimated that almost two-thirds of the individuals diagnosed with AD are women.^2^ Therapies for AD are of limited effectiveness.^3^

Gonadal hormones act as critical neurotrophic factors in the perinatal period and throughout the lifespan.^4,5^ The predilection for dementia in females suggests that there are structural and sex steroid-dependent differences between male and female brains.^6,7^ Our study seeks to establish a working framework around the reproductive axis that would allow clinicians to identify key transitions in a woman's life. The identification of key transitions along the reproductive axis could highlight critical junctures for medical intervention before the onset of cognitive decline.

## Methods

Data were collected from 289 Caucasian female patients aged 65 to 98 years for 2 years from a community-based out-patient setting in a metropolitan area of the midwest. The out-patient clinic serves both men and women and all races. Convenience sampling was used as the majority of women in the age range of interest were Caucasian. Informed written consent was obtained from all patients or their legal representatives and participation was voluntary. The collection of data was under the approval of UMKC institutional review board (16-001).

All patients presenting in this age group were offered the opportunity to participate in the survey. The survey was modified from the original design. The initial survey included age, body mass index (BMI), Fitzpatrick phototype, obstetrical history, history of cholecystectomy, presence and severity of vasomotor and sleep-related complaints, age at the final menstrual cycle, ever use of hormone replacement therapy (HRT) and duration, ever use and duration of statin medications, early adulthood or maximal height per patient recall and current height as measured in the office, maternal age at birth, number of siblings, birth order, age difference of the next older sibling, and dementia status.

History of cholecystectomy and statin use were of interest due to the importance of cholesterol oxidation to the bile salt impact on metabolism and hormones. Variables related to the presence and severity of vasomotor and sleep complaints, age at final menses, ever use and duration of HRT, and ever use and duration of statin therapy were removed due to difficulty with recall.

Maternal and sibling data were collected to function as proxy measurement of the effect of the maternal–fetal interface on the fetus. The Fitzpatrick phototyping scale was included to acknowledge the role of internal melanins in the control of inflammation in important structures in the ventral midbrain as well as the common embryologic origins of external and internal melanins.^8,9^ The scale has six levels, with the lower numbers identifying light colorations and melanin mixtures that include pheomelanin. The scale was applied by one individual to reduce observer variability. Cholecystectomy was included to recognize the role of cholesterol oxidation and bile salt governance of metabolism, hormones, and drug clearance.^10,11^

Dementia diagnosis was included if one of the following was noted: documented diagnosis of dementia in the patient's problem list, current prescription and use of medications used for Alzheimer's dementia, or endorsement of a dementia diagnosis by a caregiver present at the time of the clinic visit. Descriptors regarding dementia type, onset, and severity were not included in the study design.

Descriptive statistics were calculated for collected patient variables. The main outcome variable was dementia status; Spearman correlations were calculated between dementia status and age, BMI, Fitzpatrick skin phototype, obstetrical history, history of cholecystectomy, early adulthood or maximal height and current height, maternal age at birth, number of siblings, birth order, and age difference of the next older sibling. Simple logistic regression was conducted to estimate and test potential predictors for dementia status. All analyses were conducted using SAS 9.2.

## Results

A total of 289 female subjects were included in our study. Simple analysis showed age ranged from 65 to 98 years old, with a mean of 76 years old. Of 289 patients, 44 had evidence of mild-to-moderate cognitive decline. Spearman correlation analysis showed significant correlation between dementia status and advancing age (*r* = 0.219, *p* = 0.000), lower Fitzpatrick skin phototype (*r* = −0.141, *p* = 0.019), current height (*r* = −0.215, *p* = 0.001), and maximum height (*r* = −0.173, *p* = 0.005).

Results from the simple logistic regression model show that specific predictors of risk for dementia were advancing age (OR = 1.082 [1.034–1.132]; *p* = 0.0007], lower Fitzpatrick skin phototype 1 versus 3 (OR = 8.715 [1.124–67.562]; *p* = 0.0261), and current height (OR = 0.730 [0.614–0.866]; *p* = 0.0003). Of the four variables related to fetal origins: maternal age, number of siblings, birth order, and age difference between the subject and the next older sibling, none were found to be statistically significant. Since age is a significant predictor of risk for dementia, it was included as a covariate in the aforementioned logistic regression model (Tables 1 and 2).

**Table 1.** Descriptive Statistics on 289 Caucasian Women

Variable	N	All, N = 289	No cognitive impairment, N = 245	Cognitive impairment, N = 44
		Mean (SD) or %	Mean (SD) or %	Mean (SD) or %
Age (years)	289	77.12 (6.8)	76.66 (6.6)	80.57 (6.8)
Body mass index	285	26.93	27.145	25.54
Fitzpatrick photoype	284	1.78 (0.7)	1.81 (0.7)	1.55 (0.6)
Gravida	253	2.33 (1.6)	2.27 (1.5)	2.77 (1.9)
Para	252	2.18 (1.5)	2.12 (1.4)	2.59 (1.9)
Cholecystectomy	252	29%	28.5%	33.3%
Maternal age	229	27.33 (6.0)	27.18 (6.0)	28.13 (6.7)
Sibling No.	227	2.62 (2.14)	2.56 (2.1)	2.90 (2.4)
Birth order	218	2.17 (1.4)	2.10 (1.4)	2.68 (1.6)
Age next oldest child	122	3.97 (3.0)	3.82 (2.9)	4.24 (3.3)
Current height	243	63.13 (2.6)	63.34 (2.5)	61.48 (2.4)
Maximum height (in)	269	64.51 (2.5)	64.68 (2.5)	63.60 (2.0)
Change in height (in)	235	1.49 (1.14)	1.48 (1.1)	1.73 (1.2)

Maternal age, number of siblings, birth order, and age difference to the next oldest child were used as a proxy for the maternal–fetal interface.

**Table 2.** Simple Logistic Regression on Investigated Variables

Effect	Odds ratio	Lower CL	Upper CL
Age (years)	1.082	1.034	1.133
Body mass index	0.944	0.88	1.005
Fitz 1 vs. 3	8.715	1.124	67.562
Fitz 2 vs. 3	5.803	0.753	44.689
Gravida	1.195	0.977	1.459
Para	1.222	0.978	1.527
Cholecystectomy	0.797	0.384	1.656
Maternal age	1.026	0.963	1.09
Sibling No.	1.07	0.893	1.262
Birth order	1.268	0.988	1.61
Age next old child	1.046	0.896	1.205
Current height (in)	0.73	0.609	0.86

Fitzpatrick phototyping was included to characterize the quantity of external melanins.

## Discussion

We found statistically significant relationships with age, Fitzpatrick phototype, and current height in our search for clinical and phenotypic traits that correlate with cognitive impairment in older Caucasian women. We divided the reproductive axis into four quadrants: fetal origins, reproduction, aromatization through menopause, and beyond menopause. Surrogate clinical markers were assigned for each transition. The importance of the menopausal transition in women's health is the subject of larger and more complete studies directed to midlife and the menopausal transition.^12–18^

Biometric data were combined with number of pregnancies, BMI, delta height, history of cholecystectomy, and Fitzpatrick phototype. Cholecystectomy was included as a measure of metabolic health and an example of iatrogenic pressure on cholesterol efflux and sex steroid pathways^10,11^

### Fitzpatrick phototype

Our study used the Fitzpatrick skin phototype scale to characterize the amount of melanin pigment in the skin.^8^ Loss of pigment in the hair, skin, and eye is characteristic features of aging. Melanins differ in oxidation potential and serve important functions in the control of inflammation.^19–22^ The most prevalent melanins, eumelanin, pheomelanin, and neuromelanin, share common embryonic origins and are derived from neural crest cells.^23–25^ Pheomelanin, the prevalent melanin in fair individuals, has enhanced pro-oxidant properties and shares a common cysteinyl-dopamine isomer with neuromelanin.^26,27^ Eumelanin is the predominant melanin in darker skin individuals and has photoprotective and antioxidant properties. When coupled with iron, eumelanin functions more like pheomelanin.^27^

Neuromelanin is derived from catecholamine oxidation, and because of the presence of catechol groups in its structure, it functions as a strong chelator of heavy metals, including iron, cadmium, mercury, and lead.^28^ Heavily pigmented regions of the ventral midbrain such as the locus coeruleus (LC) and substantia nigra (SN) show pathology in AD and Parkinson's disease. Because ferritins are poorly expressed in the LC and SN neurons, neuromelanin is the major buffer system against metal toxicity in these brain regions.^29–31^

Noradrenergic neurons of the LC express estrogen receptors, implicating estrogen as a key modulator of noradrenergic signaling within the brain.^32^ Estrogen exerts opposite effects on tyrosine hydroxylase activity, the rate-limiting step in catecholamine synthesis, in the LC in male and female brains.^32,33^ In contrast, sex-dependent effects of estrogen on noradrenergic signaling is an interesting component of AD. We hypothesize that changes in external melanins may reflect change in internal melanins. There may be phenotypes related to cysteine availability and tyrosinase stability that reflect selective pressure across time and race.

Age is the most significant risk factor for AD.^1,34^ Aging leads to depletion of sex hormones due to gonadal atrophy.^35,36^ Although women experience an abrupt loss of ovarian sex hormones after menopause and thus significant disruption to the hypothalamic-pituitary-ovarian axis, bioavailable testosterone in men declines by only 2% to 3% per year after 30 years.^2^

### Change in height as a proxy for estrogen deficiency

Our study uses delta height, or the difference between maximal reported height and current height, as a clinical marker for estrogen deficiency. Normally, estrogen directly inhibits the function of osteoclasts.^37^ Loss of estrogen correlates with loss of mechanisms for bone maintenance.^38,39^ Postmenopausal women commonly suffer from osteopenia and osteoporosis due to these changes, which can manifest in loss of height.

### Areas for future research

A disease as complex as AD may represent a stacking of variables across a lifetime, some of which may provide opportunities for intervention before the onset of overt cognitive decline. Advances in maternal–fetal health have highlighted the gestational environment as a key programming juncture for both the mother and the child. The view of women's health as a continuum across the reproductive timeline should have firm grounding in medical school curricula.

### Limitations

Our study includes only Caucasian female subjects, geographically limited to urban and rural areas surrounding Kansas City, Missouri. The small study size will limit generalizability. The breadth of our data collection was limited by patient recollection and lack of recorded data. The menopausal transition was difficult to isolate as many women in this age group had partial or total hysterectomies before they would have gone through natural menopause. Conjugated estrogen use was common until the early 2000s.

## Conclusion

Our study suggests that age, lower Fitzpatrick phototype, and age-related decrease in height are associated with Alzheimer's dementia. Future research should further explore the hormonal and physiological changes that may program AD risk in women. The concept of dementia risk as an accrual process across the reproductive axis may foster shared goals between obstetrics, pediatrics, and internal medicine. The validity of this concept, that dementia in women may require a broad view of the reproductive axis, requires further study.

## Abbreviations Used

AD: Alzheimer's disease
HRT: hormone replacement therapy
LC: locus coeruleus
SN: substantia nigra
